# Development and Initial Psychometric Evaluation of the Post-Acute Acuity Rating for Children

**DOI:** 10.1155/2015/841523

**Published:** 2015-11-02

**Authors:** Jane E. O'Brien, Helene M. Dumas

**Affiliations:** ^1^Franciscan Hospital for Children, 30 Warren Street, Boston, MA 02135, USA; ^2^Research Center for Children with Special Health Care Needs, Franciscan Hospital for Children, 30 Warren Street, Boston, MA 02135, USA

## Abstract

The Post-Acute Acuity Rating for Children (PAARC) is the first known acuity rating intended to reflect medical severity based on age, reason for admission, diagnoses, dependence in activities of daily living, and technology reliance for children admitted to post-acute care rehabilitation hospitals. Content validity was tested using an expert panel scoring the Content Validity Index (CVI). Concurrent validity was examined using clinician's opinion of acuity at admission, the Complexity Index, and All Patient Refined Diagnosis Related Group (APR-DRG) codes. Predictive validity was examined with acute care readmission within 30 days. Interrater reliability was assessed using admission histories from closed cases. Content validity was established and concurrent validity was moderate to high with clinician opinion (rho = .76, *p* < .001), the Complexity Index (rho = .76, *p* < .001), and APR-DRGs (rho = .349, *p* = .001). Predictive validity was moderate (rho = .504, *p* = .005) and returns to acute care within 30 days. Interrater reliability was excellent (ICC = 0.97; 95% CI = 0.92–0.90, *p* < .001). Experts agreed that the PAARC's content is relevant, simple, and representative of the population. The PAARC measured well against indicators of medical complexity for pediatric outpatient care and medical record coding and was reliable between raters. This work supports proceeding with additional development and validity testing of the PAARC.

## 1. Introduction

Children are most often admitted to a pediatric post-acute care rehabilitation hospital following acute care hospitalization but are not united by a common medical diagnosis, a mutual type of technology dependence, or quantification of medical care needs [1]. Acuity ratings exist for different levels of hospital care, including acute [2] and emergency care [3], and provide an indication of patient severity. Patient acuity ratings help hospitals to delineate care provider roles, employ evidence-based practice, promote efficiency, and evaluate effectiveness [2–7]. No acuity measures exist for pediatric post-acute rehabilitation hospital care, however, making it difficult to accurately evaluate the effectiveness of clinical practice and research.

The Post-Acute Acuity Rating for Children (PAARC) is a new 11-level acuity rating intended to provide a simple, uniform measure for children admitted to a post-acute care rehabilitation hospital. The PAARC was originally presented as “Clinical Program Groups” (noncomplex infants, medically complex infants and children, active rehabilitation, and ventilator (noninvasive and invasive)) and, in pilot work, it was noted that these Clinical Program Groups differed by age, admission number, discharge disposition, and total admission reimbursement [1]. In additional work, these Clinical Program Groups were identified as a primary contributing factor along with age (younger) and length of time in the post-acute care hospital (<30 days) for prediction of an unplanned readmission to acute care [8]. These studies confirmed the need for further development of a severity rating scale to provide a common metric for clinical documentation, communication, and research in pediatric post-acute rehabilitation hospital care.

The PAARC's rating options reflect a child's level of medical severity based on age, reason for post-acute care hospital admission, diagnoses, and technology dependence. The levels of the PAARC are intentionally broad and not intended to describe all aspects of care and/or function for individual children. Children's acuity is rated at the highest level described on the scale and children can move between levels as the PAARC is intended to measure acuity at the present time. The PAARC does not identify the cause of illness, the clinical prognosis for an individual child, an estimated length of hospital stay, or direct care provision.

A new scale such as the PAARC requires an examination of its psychometric properties including validity and reliability to ensure the confidence of clinicians and researchers in its utility and effectiveness [9]. The purpose of this study was to examine the content, concurrent and predictive validity, and interrater reliability of the PAARC.

## 2. Methods

Clinical and research staff at Franciscan Hospital for Children (FHC), Boston, MA, a pediatric post-acute care rehabilitation hospital, developed the PAARC based on a literature review, clinical experience, and a review of existing pediatric and adult acuity measures [2, 3, 6, 7, 10] and pediatric functional classification systems such as the Gross Motor Functional Classification System (GMFCS) [11] and the Manual Classification Ability Classification System (MACS) [12]. The Institutional Review Board at FHC reviewed and approved this study.

### 2.1. Content Validity

Content validity of the PAARC was evaluated in 2 phases with expert review using the Content Validity Index (CVI) [13] (Table 1). The CVI provides evidence of content validity by computing a score using ratings of relevance, accuracy, simplicity, and clarity by content experts [13, 14]. In Phase 1 (January-February 2014), an email was sent to 5 experts (physicians and pediatric nurse practitioners) in the care of children with medical complexity and pediatric post-acute hospital care known to the study authors through previous research collaboration that introduced the study objective and requested assistance in reviewing, scoring, and commenting on the PAARC via an online survey link. In addition to the CVI scores, experts were asked to respond to a question about the PAARC's representativeness of a post-acute care rehabilitation hospital pediatric population (“Is this acuity rating representative of a post-acute care hospital pediatric population?”) providing a yes/no response. A reminder was sent via electronic mail after 2 weeks to encourage participation. The survey results remained anonymous. Three of the 5 experts (67%) completed the CVI and provided comments and 1 expert who did not complete the CVI provided written comments via email. The PAARC was revised based on Phase 1 feedback. The revisions included the addition of 3 acuity levels, the inclusion of additional examples of specific conditions, technology, and care needs, and minor wording changes.

For Phase 2 (March 2014), the revised PAARC was sent via email to 8 experts identified via previous collaboration with the study authors, who were asked to score the CVI and comment on the PAARC using a link to an online survey. In addition to the CVI scores, the question regarding the PAARC's representativeness of a post-acute care hospital pediatric population was retained. Phase 2 experts included the 5 experts from Phase 1 and additional 3 experts, also medical doctors, physician assistants, and/or nurse practitioners with expertise in the care of children with medical complexity and pediatric post-acute hospital care. A reminder was sent via electronic mail after 2 weeks to all potential respondents to encourage participation of those who had not responded, so that responses could remain anonymous. Six experts (75%) completed the CVI in Phase 2 and provided comments.

### 2.2. Concurrent Validity-Clinician Opinion, Complexity Index, and APR-DRG

To assess concurrent validity of the newly developed PAARC (Table 3), from July 2014 to October 2014, an admitting medical staff member (medical doctor (MD), physician assistant (PA), or nurse practitioner (NP)) completed the PAARC for a consecutive sample of 44 admissions to Franciscan Hospital for Children, a pediatric post-acute rehabilitation hospital. In addition, the clinician completed the following: (1) a 5-point Likert scale (strongly disagree to strongly agree) rating their perception of the child's acuity at admission and (2) the Complexity Index [10], a 10-level measure used by primary care providers in medical home models to classify children and youth with special heath care needs based on their medical severity and the social or family complexity of a child's condition (see Appendix).

To further examine concurrent validity of the PAARC, PAARC scores were compared with All Patient Refined Diagnosis Related Groups (APR-DRGs) scores, a computerized scoring system used in United States hospitals to adjust patient data for severity of illness and risk of mortality. The 3M APR-DRGs software assigns patients to severity and mortality subclasses considering patient age, medical procedures, and principal diagnosis. There are 315 base APR-DRGs (version 27.0) and each APR-DRG is subdivided into four severity of illness subclasses and four risk of mortality subclasses [15]. PAARC ratings were assigned retrospectively by the study authors using the FHC admission history and physical in the electronic medical record for 82 children discharged consecutively from FHC from October 1, 2013, to March 31, 2014.

### 2.3. Predictive Validity

To determine the predictive validity of the PAARC for forecasting a child's return to acute care within 30 days of admission to post-acute care, the attending clinician completed the PAARC using the child's admission history and physical (as described previously) and replied to 1 question on a 5-point Likert scale assessing the likeliness of readmission to acute care within 30 days of their admission to the post-acute care hospital (very unlikely to very likely). The clinician then also selected from a list of the potential clinical reasons for readmission for each child (see Appendix). Once a child was discharged, additional demographic data was collected from the child's electronic medical record including date of birth, gender, admitting diagnoses, and discharge disposition by the study's authors.

### 2.4. Interrater Reliability

Six medical staff members (2 MDs, 1 PA, and 3 NPs; 1 male and 5 females) from Franciscan Hospital for Children rated 10 cases using the PAARC to assess interrater reliability. The six medical staff members were selectively recruited and introduced to the PAARC by the authors, written consent was obtained for participation, and 2 practice cases were completed as a group. The rationale for the ratings for each of the 2 practice cases was discussed as a group before each participant proceeded to rate 10 discharged cases (deidentified for child and documenting clinician) from the pediatric post-acute care rehabilitation hospital inpatient admission history and physical report. The clinicians were instructed to review the admission history and physical for each case, not to consult with each other, and provide a score on the provider score sheet. Each staff member was assigned a rater number, and score sheets were collected for analysis by the authors.

The children whose records were used for the 2 practice cases and 10 cases for analysis ranged in age from 6 days to 14 years of age and included 7 females and 5 males. PAARC ratings spanned PAARC levels 1–5. Examples of post-acute care hospital admission diagnoses for these cases included neonatal abstinence syndrome; multiple congenital anomalies (with poor feeding tolerance and weight gain, s/p gastrostomy tube placement); acquired brain injury; prematurity with chronic lung disease and pulmonary hypertension; and degenerative neuromuscular disorder with new ventilator dependence.

### 2.5. Statistical Analysis

Content validity was assessed by calculating a CVI for relevance, clarity, simplicity, and ambiguity for the PAARC. The CVI is a calculation of the proportion of total items given a “positive” rating (a “3” or “4” on a 1 to 4 scale) with a CVI score of 1.00 recommended for <3 respondents [14] and 0.8 or higher considered acceptable with >3 respondents [16].

The concurrent validity of the PAARC was assessed with the clinician opinion (Likert scale) rating scores, Complexity Index [10] scores, and APR-DRG [15] scores using Spearman correlations to estimate and detect significant associations. The predictive validity of the PAARC was assessed by correlating the PAARC score with the discharge disposition (home/planned transfer to acute care versus unplanned transfer to acute care) using a Spearman correlation. Correlations were deemed moderate if rho = 0.3 to 0.5 and strong if > 0.5. To assess interrater reliability, intraclass correlation coefficients ICC_2,1_ were generated and percent agreement of the raters for the 10 cases against a preestablished key was calculated.

## 3. Results

### 3.1. Content Validity of the PAARC

In Phase 1 assessment of content validity, the percentage of raters who judged the PAARC as valid reached 1.00 only for relevance. Comments included recommendations to (1) revise the scale description to include instruction to rate each child at the highest level; (2) further break down the levels to distinguish the number and needs for a child; (3) add examples of medical conditions, diagnoses, and technology use; and (4) distinguish infants from children and youth for all levels. In Phase 2, the proportion of raters who judged the PAARC as valid exceeded 80% for relevance and simplicity. Multiple experts questioned whether children can be assigned different levels if changes occur over time and additional suggestions were made to further clarify the objective of the scale and its instructions. Table 1 displays the proportion for each CVI for Phases 1 and 2. For the additional question of representativeness of a post-acute care hospital pediatric population, for both Phase 1 and Phase 2, there was 100% agreement amongst the expert raters.

### 3.2. Concurrent Validity with Clinician Opinion, Complexity Index, and APR-DRGs

Characteristics of each sample for the concurrent and predictive validity and interrater reliability analyses are presented in Table 2. A strong and statistically significant correlation (rho = .76, *p* < .001) was found between the PAARC and clinician opinion of acuity and between the PAARC and the Complexity Index [10] (rho = .78, *p* < .001) for a sample of 44 post-acute care hospital admissions from July, 2014, to December, 2014. A moderate but significant correlation (rho = .349, *p* = .001) was found between the PAARC and APR-DRG scores for 82 discharges from October, 2013, to March, 2013.

### 3.3. Predictive Validity of the PAARC

Admission and discharge data were available for 30 infants and children for an analysis of predictive validity as 14 children were still in the post-acute care hospital at the time of study completion (December, 2014). Fifteen children had planned discharges (home or planned discharge to acute care for a scheduled procedure) and 15 had an unplanned discharge to acute care due to an acute medical need (e.g., respiratory or cardiac instability). At admission, clinicians predicted that 15 of the 30 children discharged were likely or very likely to have an unplanned readmission to acute care. Reasons for predicting likelihood of an unplanned readmission included one or a combination of the following reasons: technology dependence, number and/or type of medications, dependence in activities of daily living (e.g., feeding and mobility), cardiac instability, infection, and/or a GI concern. For the 15 children who had an unplanned discharge, clinicians predicted correctly for 11 of the 15 (73%) cases, were undecided for 2 (13%) of these cases, and predicted incorrectly for 2 cases (13%). The correlation between the PAARC and discharge disposition (planned or unplanned discharge) was moderate and significant (rho = .504, *p* = .005).

### 3.4. Interrater Reliability of the PAARC

Excellent interrater reliability (intraclass correlation coefficient [ICC], 0.97; 95% confidence interval [CI], 0.92–0.90, *p* < .001) was demonstrated. For the individual cases, percent agreement among the raters ranged from 67% to 100% for each of the 10 cases.

## 4. Discussion

No measure of acuity currently exists for post-acute rehabilitation hospital care in pediatrics. The purpose of this study was to introduce and report validity and reliability of the newly developed Post-Acute Acuity Rating for Children (PAARC). In this study, the PAARC was determined to be representative of a pediatric post-acute care rehabilitation hospital population, relevant and simple, but required revision to be clear and unambiguous. The PAARC demonstrated a moderate to high degree of concurrent validity and a high degree of agreement between raters.

The determination of a scale's content validity must indicate that a measure is serving its intended purpose. Content validity is a measure of the degree to which a scale represents an adequate operational definition of a concept [17, 18] and is often established through a review of the existing literature and expert evaluation and is often appraised in terms of relevance, clarity, simplicity, and ambiguity [9, 13]. Content validity requires that the scale developers have judiciously selected the experts to provide the rating while it is understood that more than one round of feedback may need to be solicited [16]. The process in this study adhered to these principles as the PAARC was developed based on clinical experience, current (though limited) research evidence, and preliminary research of pediatric post-acute care clinical groupings identifying differences for the levels of the scale [1]. The expert panel in this study had representatives from multiple pediatric acute and post-acute care rehabilitation hospitals were of varying professions and representative of staff with administrative, clinical, and research experience with the target population.

We attempted to strengthen and evaluate the establishment of content validity by using the CVI. The CVI has been criticized, however, for its focus on item relevance of only the items reviewed and its inability to capture whether a scale includes a comprehensive set of items to adequately measure the construct of interest [17, 18]. By soliciting open-ended comments and feedback along with the CVI ratings, this was addressed.

An Item-CVI (I-CVI) using ratings of individual items may be computed or, alternatively, a Scale-CVI (S-CVI) may be computed. Although the PAARC has multiple levels, it is essentially a 1-item scale (the clinician chooses 1 “level” for a child). Thus, with only one item, there is no distinction to be made in this study. It has been recommended that 3 to 6 experts be used for the CVI and suggested that when 6 or more experts are used to evaluate the CVI, a minimum I-CVI of 0.78 is required, and S-CVI/Ave of 0.90 should be the minimum value for retaining items and supporting scale content validity [14, 16]. Though the recommended proportion of 0.8 was reached only for relevance and simplicity in Phase 2, we chose to make the suggested revisions and proceed with interrater reliability and validity testing as the suggestions were deemed minor and easily addressed. While we chose to examine relevance, clarity, simplicity, and ambiguity, it has also been noted that only relevance and clarity may be assessed, while others assess relevance only [16–18].

Concurrent validity is determined by examining the extent to which a score on a scale or test predicts scores on a well-established criterion measure. Examining concurrent validity was challenging as there is no “gold standard” against which to compare the PAARC. We used clinician perception as a concurrent measure as clinician ratings have been used in measurement validation research for concurrent validity previously [19]. We also used the Complexity Index [10] which was developed for use by primary care providers in medical home models to classify children and youth with special heath care needs based on their medical severity and the social or family complexity of a child's condition. Lastly, we examined the relationship between the PAARC and APR-DRGs, for which there are no known previous reports.

As a measure of severity, predictive validity for future occurrences such as hospital length of stay and/or readmission to acute care is extremely important. Predictive validity is examined when scores of the new test are compared with results obtained at some point in the future. In this study, the predictive validity of the PAARC was high and statistically significant. Of note, clinicians were accurate almost 75% of the time in predicting an unplanned readmission to acute care.

Interrater reliability represents the degree to which different judges agree in their ratings and is likely strengthened by clear guidelines and instructions [20]. The scores of the raters in this study were highly reliable, suggesting that it is possible for different raters to consistently use the PAARC to rate level of acuity for infants and children admitted to a post-acute care hospital. These scores also indicate that the instructions and guidelines used to introduce and explain the PAARC to the clinicians were successful.

There are limitations with this study that reduce the generalizability of the results. For example, while soliciting expert feedback is a common strategy for establishing content validity, feedback is subjective and likely to exhibit bias (intentional or unintentional) based on previous professional experiences, current clinical setting, and rater expectancy. In addition, the excellent interrater reliability was achieved with clinicians from only 1 facility who may be predisposed to “think alike.” Sample sizes for each phase of the reported evaluations of the PAARC's psychometric properties were small; however, statistically significant results were obtained.

## 5. Conclusion

The PAARC is the first known acuity measure for children in post-acute care. Validity and reliability were established for the PAARC for use in a post-acute pediatric rehabilitation hospital setting. Given these results, it is appropriate to proceed with further evaluation of the PAARC's clinical feasibility, usefulness in clinical practice, and further evaluation of its psychometric properties, as the PAARC has the potential to serve as a common descriptor of children in much-needed multisite studies in pediatric post-acute care.

## Figures and Tables

**Table 1:** Content Validity Index and results.

	Ratings				Phase 1^*∗*^	Phase 2^*∗*^
	1	2	3	4		
Relevance	Not relevant	Needs some revision	Relevant but needs minor revision	Very relevant	**1.00**	**0.83**
Clarity	Not clear	Needs some revision	Clear but needs minor revision	Very clear	0.67	0.67
Simplicity	Not simple	Needs some revision	Simple but needs minor revision	Very simple	0.67	**0.83**
Ambiguity	Doubtful	Needs some revision	No doubt but needs minor revision	Meaning is clear	0.67	0.67

^*∗*^Proportion of expert ratings = 3 or 4 by participating experts.

**Table 2:** Characteristics of samples for concurrent and predictive validity analyses.

	* *Concurrent validity		Predictive validity
	Clinician opinion and Complexity Index	APR-DRGs	
Total sample (*n*)	44	82	30
Gender (males, %)	25 (57%)	37 (45%)	17 (57%)
Age at admission (years)^*∗*^	5.62 (6.62)	3.68 (5.30)	5.08 (6.53)
	0.03–21.52	0.01–20.99	0.03–19.1
Age at discharge (years)^*∗*^	N/A	3.85 (5.28)	5.67 (6.52)
		0.07–21.12	0.7–19.18
Length of stay in post-acute care (days)^*∗*^		61.24 (70.78)	33.33 (20.72)
		4–400	6–74
PAARC levels			
Level 1.1 (noncomplex infant)	2 (5%)	10 (12%)	2 (7%)
Level 1.2 (noncomplex child)	2 (5%)	0 (0%)	1 (3%)
Level 2 (active rehabilitation)	2 (7%)	16 (20%)	0 (0%)
Level 3.11 (medically complex infant: 1-2 systems)	4 (9%)	3 (4%)	4 (13%)
Level 3.12 (medically complex infant: 3+ systems)	2 (5%)	14 (17%)	1 (3%)
Level 3.21 (medically complex child: 1-2 systems)	6 (14%)	2 (2%)	5 (17%)
Level 3.22 (medically complex child: 3+ systems)	3 (7%)	5 (6%)	2 (7%)
Level 4.1 (noninvasive ventilation infant)	5 (11%)	7 (9%)	3 (10%)
Level 4.2 (noninvasive ventilation child)	2 (5%)	0 (0%)	2 (7%)
Level 5.10 (invasive ventilation infant)	4 (9%)	13 (16%)	4 (13%)
Level 5.20 (invasive ventilation child)	11 (25%)	12 (15%)	6 (20%)

^*∗*^Mean (SD) range.

**Table 3:** 

Level		Description
1.1 Noncomplex infant	Infant (<1 year) Medically stable	*Medically stable infant* (<1 year of age) with special health care needs admitted to the hospital for medication weaning and/or feeding, growth, and development issues; infant's function is below age-appropriate and care is more complex than what is typically required for an infant

1.2 Noncomplex child	Child/youth (>1 year) Medically stable	*Medically stable child or youth* from 1 to 21 years of age who requires hospitalization; child's function is below age-appropriate and medical care is needed that is not typically required for children, such as postsurgical management, IV antibiotics, and wound care

2 Active rehabilitation	Child/youth (>1 year) Medically stable Active rehabilitation therapies	*Medically stable child or youth* 1 to 21 years of age who will *receive active level of rehabilitation therapies* including physical, occupational, and/or speech-language therapy; child may or may not have a preexisting condition but exhibits medical needs and impairments in self-care, safety, communication, cognition, behavior, and/or mobility due to a recent illness/injury/surgery

3.11 Medically complex infant	Infant (<1 year) Medically complex 1-2 major systems	*Infant* with a chronic condition/special health care need who *requires medical care and support for 1-2 major systems* such as tube feeding, total parenteral nutrition, IV antibiotics, wound care, musculoskeletal traction, nasal cannula oxygen, and tracheostomy care (no ventilator)

3.12 Medically complex infant	Infant (<1 year) Medically complex 3+ major systems	*Infant* with a chronic condition/special health care need who *requires medical care and support for 3 or more major systems* such as tube feeding, total parenteral nutrition, musculoskeletal traction, nasal cannula oxygen, and tracheostomy care (no ventilator)

3.21 Medically complex child	Child/youth (>1 year) Medically complex 1-2 major systems	*Child or youth* with a chronic condition/special health care need who *requires medical care and support for 1-2 major systems* such as tube feeding, total parenteral nutrition, musculoskeletal traction, nasal cannula oxygen, and tracheostomy care (no ventilator)

3.22 Medically complex child	Child/youth (>1 year) Medically complex 3+ major systems	*Child or youth* with a chronic condition/special health care need who *requires medical care and support for 3 or more major systems* such as tube feeding, total parenteral nutrition, musculoskeletal traction, nasal cannula oxygen, and tracheostomy care (no ventilator)

4.1 Noninvasive ventilation infant	Infant (<1 year) Noninvasive ventilation	*Infant* (<1 year of age) who requires *noninvasive* (*no tracheostomy*) *respiratory support* (e.g., continuous positive airway support) for survival for any amount of time during a 24-hour period

4.2 Noninvasive ventilation child	Child/youth (>1 year) Noninvasive ventilation	*Child or youth* who requires *noninvasive* (*no tracheostomy*) *respiratory support* (e.g., continuous positive airway support) for survival for any amount of time during a 24-hour period

5.1 Invasive ventilation infant	Infant (<1 year) Invasive ventilation	Infant (<1 year of age) who requires *invasive* (*tracheostomy*) *respiratory support* (positive pressure mechanical ventilation) for survival for any amount of time during a 24-hour period

5.2 Invasive ventilation child	Child/youth (>1 year) Invasive ventilation	Child or youth who requires *invasive* (*tracheostomy*) *respiratory support* (positive pressure mechanical ventilation) for survival for any amount of time during a 24-hour period

## Appendix

### Clinician Opinion of Acuity Likert Scale, Complexity Index, and Clinician Opinion of Readmission to Acute Care

*Likert Scale: Clinician Opinion of Acuity*. In your opinion, at admission to Franciscan, this child has a* high level of acuity*. (Please circle one)

Strongly Disagree
Disagree
Undecided
Agree
Strongly Agree

Please assign a* Complexity Index Score*. (Place a check in the appropriate box).

□ Well child
□ Well, no medical problems, but does have complicating social or family issues
□ One moderate medical problem involving one organ system
□ One moderate or severe medical problem, involving one organ system with complicating social or family issues
□ One moderate or severe medical problem, involving one organ system with complications
□ One moderate or severe medical problem, involving one organ system with complications and with complicating social or family issues
□ Two or more moderate or severe medical problems, involving two or more organ systems
□ Two or more moderate or severe medical problems, involving two or more organ systems and with complicating social or family issues
□ Two or more moderate or severe medical problems, involving two or more organ systems with complications
□ Two or more moderate or severe medical problems, involving two or more organ systems with complications and with complicating social or family issues

*Likert Scale: Clinician Opinion of Likelihood of Unplanned Readmission to Acute Care*. In your opinion, how likely is this child to have an* unplanned readmission to acute care within 30 days* of admission to Franciscan? (Please circle one)

Very Unlikely
Unlikely
Undecided
Likely
Very Likely

## Abbreviations

PAARC: Post-Acute Acuity Rating for Children
CVI: Content Validity Index
APR-DRGs: All Patient Refined Diagnosis Related Groups.
